# Supplementary material for: Population Dynamics of Epithelial-Mesenchymal Heterogeneity in Cancer Cells
Source: Biomolecules. 2022 Feb 23;12(3):348. doi: 10.3390/biom12030348 (PMC8945776; doi:10.3390/biom12030348)
Supplement: Supplementary file 1 [file biomolecules-12-00348-s001.zip › biomolecules-1586211-supplementary.pdf]

Supplementary figures

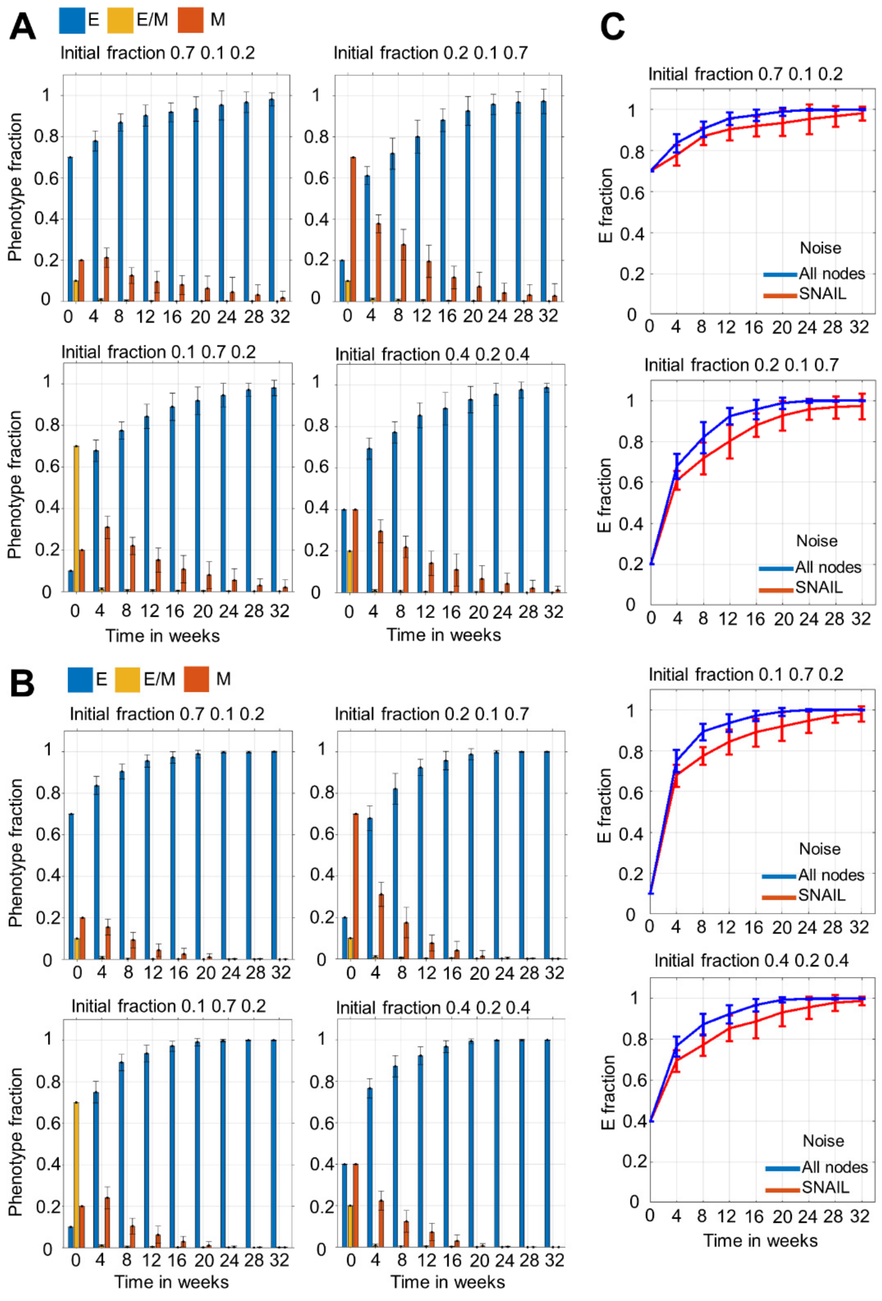

Figure S1. ***Longer time simulations and similarities between effects of fluctuation in SNAIL and all players in core EMT network during cell division.*** *(**A**) Extension of simulation of results shown in Fig1C to 32 weeks. (**B**) Temporal changes in phenotypic distribution for same initial fractions as in Fig1A but with stochastic fluctuations considered in all players of core EMT network during cell division. (**C**) Overlap in temporal changes in E fraction when SNAIL (red), and all players of core EMT network (blue) had fluctuations in their levels during cell division. Avg. doubling time (DT) of each phenotype is set to 20 hrs and scaling factors η_1 and η_2 to 0.2 and 0.1, respectively. The initial population size was 200 cells. Mean and standard deviation calculated from 16 independent runs.*

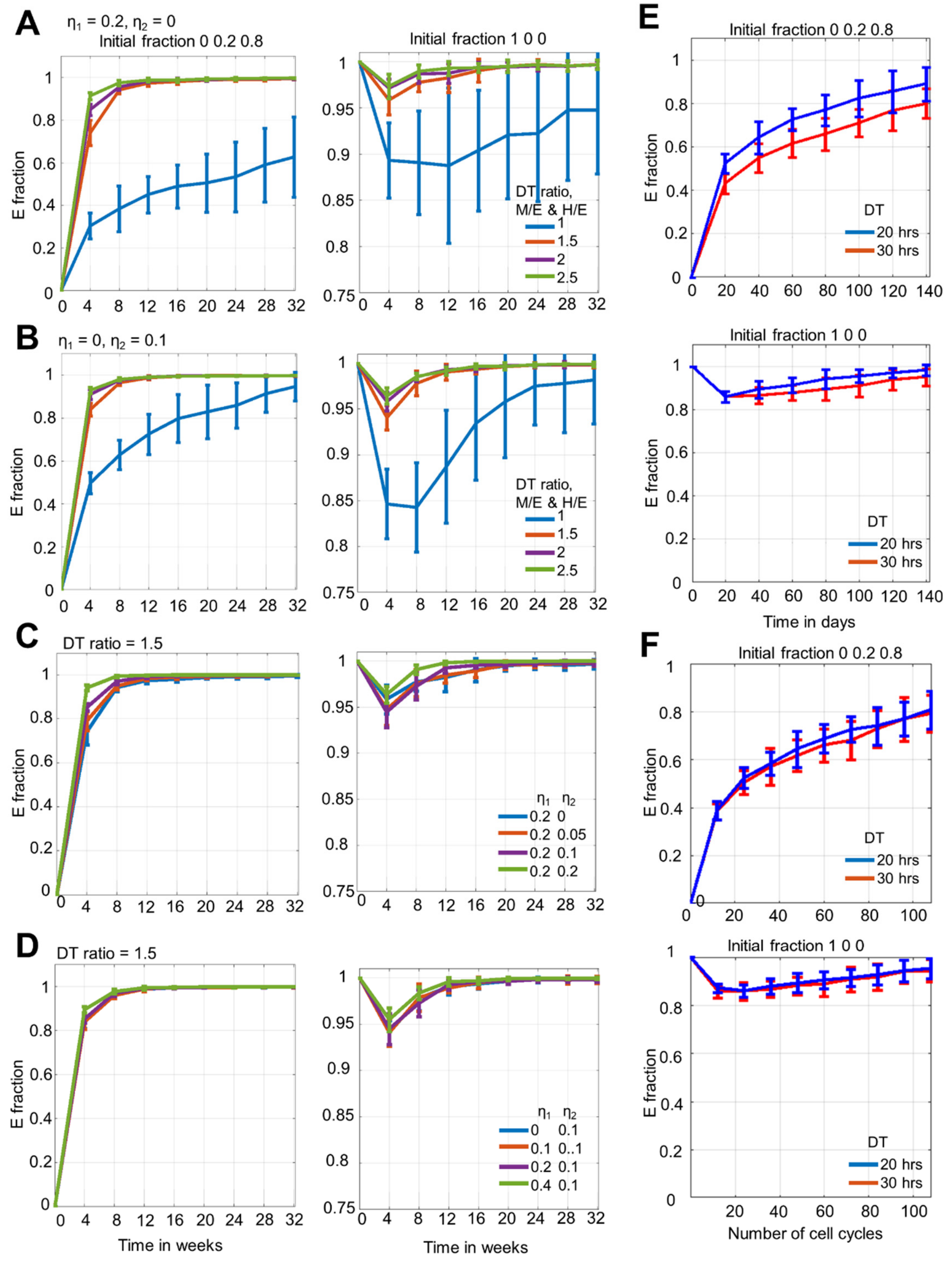

Figure S2. ***Temporal changes in E fraction for combinations of avg. doubling time (DT) ratio, η_1, and η_2 values; and higher cell doubling time.*** *Temporal E fraction changes for different DT ratios when* ***(A)*** *η_1 = 0.2 and η_1 = 0, and* ***(B)*** *η_1 = 0 and η_1 = 0.1. Temporal E fraction changes for different η_1 and η_2 values when* ***(C)*** *η_1 = 0.2 and η_2 varying, and* ***(D)*** *η_1 = varying and η_2 = 0.1. Here, the DT(E, E/M, M) = 20 hrs.* ***(E)*** *Overlap in E fraction changes for DT(E, E/M, M) = 20 hrs and DT(E, E/M, M) = 30 hrs when plotted against time in days.* ***(F)*** *Overlap in E fraction changes for DT(E, E/M, M) = 20 hrs and DT(E, E/M, M) = 30 hrs when plotted against number of cell cycles, determined by DT. In all, initial fractions of 1) Mix of E/M and M, and 2) pure E phenotypes are considered. The initial population size was 200 cells. Mean and standard deviation calculated from 16 independent runs.*

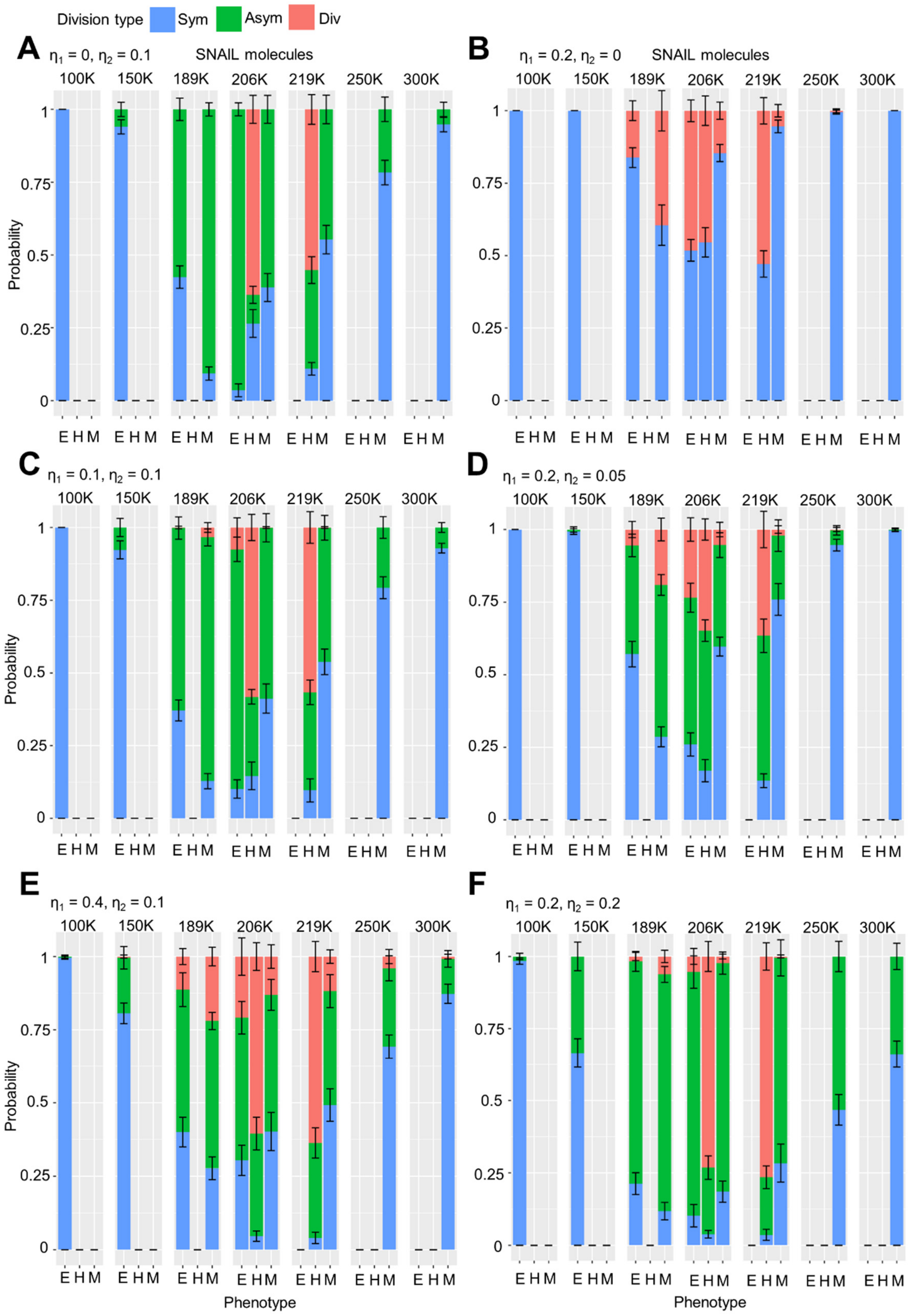

Figure S3. ***Phenotypic switching probability for various scaling factors (η_1 and η_2) values across SNAIL levels.*** *Mean, and standard deviation are calculated from 10 independent runs of 100 iterations each.*

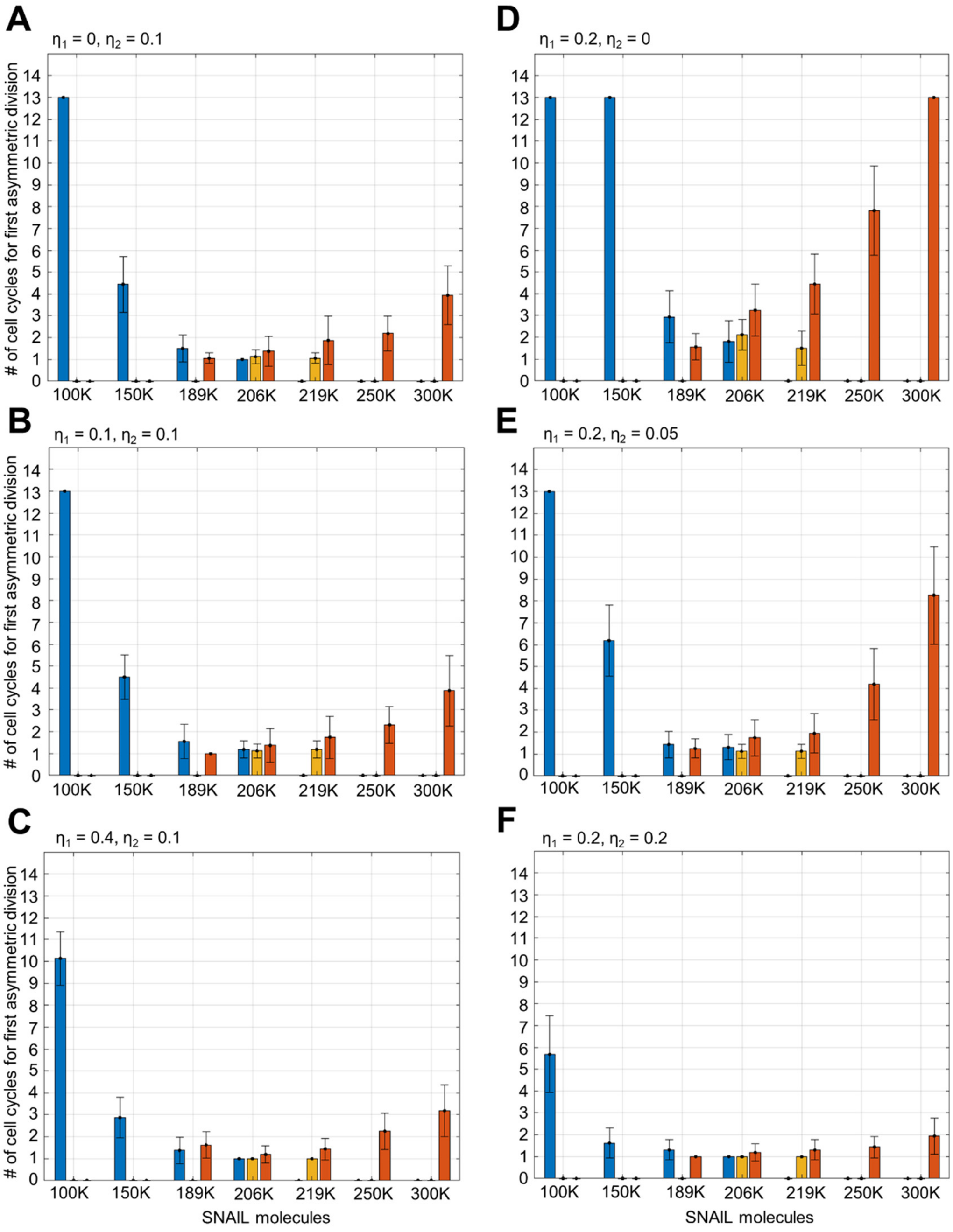

Figure S4. ***Average cell cycles/generations required for first asymmetric switching for various scaling factors (η_1 and η_2) values across SNAIL levels.*** *Progeny up to 12 generations/cell cycles were observed for phenotypic switching. Mean, and standard deviation are calculated from 16 independent runs.*

Phenotype E

SNAIL = 50K

η_1	0	0.1	0.2	0.4
0			0	1
0.1			0	1
0.2	0	0	0	1
0.4	1	1	1	0

SNAIL = 100K

η_1	0	0.1	0.2	0.4
0	0	0	0	1
0.1	0	0	0	1
0.2	0	0	0	1
0.4	1	1	1	0

SNAIL = 189K

η_1	0	0.1	0.2	0.4
0	0	0	0	0
0.1	0	0	0	0
0.2	0	0	0	0
0.4	0	0	0	0

SNAIL = 206K

η_1	0	0.1	0.2	0.4
0		0	0	
0.1	0	0	0	0
0.2	0	0	0	0
0.4		0	0	

Phenotype E/M

SNAIL = 206K

η_1	0	0.1	0.2	0.4
0	0	0	0	0
0.1	0	0	0	0
0.2	0	0	0	0
0.4	0	0	0	

SNAIL = 219K

η_1	0	0.1	0.2	0.4
0	0	0	0	0
0.1	0	0	0	0
0.2	0	0		
0.4	0	0		

Phenotype M

SNAIL = 189K

η_1	0	0.1	0.2	0.4
0	0	0	0	1
0.1	0		0	1
0.2	0	0	0	1
0.4	1	1	1	0

SNAIL = 206K

η_1	0	0.1	0.2	0.4
0	0	0	0	0
0.1	0	0	0	0
0.2	0	0	0	0
0.4	0	0	0	0

SNAIL = 219K

η_1	0	0.1	0.2	0.4
0	0	0	0	0
0.1	0	0	0	0
0.2	0	0	0	0
0.4	0	0	0	0

SNAIL = 250K

η_1	0	0.1	0.2	0.4
0	0	0	0	0
0.1	0	0	0	0
0.2	0	0	0	0
0.4	0	0	0	0

SNAIL = 300K

η_1	0	0.1	0.2	0.4
0	0	0	0	0
0.1	0	0	0	0
0.2	0	0	0	0
0.4	0	0	0	0

Figure S5. ***Statistical analysis of differences in minimum cell cycles for required asymmetric division by a cell of given phenotype and SNAIL level on varying η_1 and keeping η_2 fixed (0.1).*** *Here, 1 represents a statistically significant difference, while 0 denotes insignificance, according to p-value of 0.05. The blank space corresponds to nan (not-a-number) values resulted as an outcome of non-variability in data in and across pair of observations.*

Phenotype E

SNAIL = 50K

η_2	0	0.05	0.1	0.2
0			0	1
0.05			0	1
0.1	0	0	0	1
0.2	1	1	1	0

SNAIL = 100K

η_2	0	0.05	0.1	0.2
0		1	1	1
0.05	1	0	1	1
0.1	1	1	0	1
0.2	1	1	1	0

SNAIL = 189K

η_2	0	0.05	0.1	0.2
0	0	1	1	1
0.05	1	0	0	0
0.1	1	0	0	0
0.2	1	0	0	0

SNAIL = 206K

η_2	0	0.05	0.1	0.2
0	0	0	1	1
0.05	0	0	0	1
0.1	1	0	0	0
0.2	1	1	0	

Phenotype E/M

SNAIL = 206K

η_2	0	0.05	0.1	0.2
0	0	1	1	1
0.05	1	0	0	1
0.1	1	0	0	0
0.2	1	1	0	0

SNAIL = 219K

η_2	0	0.05	0.1	0.2
0	0	1	1	1
0.05	1	0	0	1
0.1	1	0	0	0
0.2	1	1	0	0

Phenotype M

SNAIL = 189K

η_2	0	0.05	0.1	0.2
0	0	0	1	1
0.05	0	0	0	1
0.1	1	0	0	0
0.2	1	1	0	

SNAIL = 206K

η_2	0	0.05	0.1	0.2
0	0	1	1	1
0.05	1	0	0	1
0.1	1	0	0	0
0.2	1	1	0	0

SNAIL = 219K

η_2	0	0.05	0.1	0.2
0	0	1	1	1
0.05	1	0	0	1
0.1	1	0	0	0
0.2	1	1	0	0

SNAIL = 250K

η_2	0	0.05	0.1	0.2
0	0	1	1	1
0.05	1	0	1	1
0.1	1	1	0	1
0.2	1	1	1	0

SNAIL = 300K

η_2	0	0.05	0.1	0.2
0		1	1	1
0.05	1	0	1	1
0.1	1	1	0	1
0.2	1	1	1	0

Figure S6. ***Statistical analysis of differences in minimum cell cycles required for asymmetric division for a cell of given phenotype and SNAIL level on keeping η_1 fixed (0.2) and varying η_2.*** *Here, 1 represents a statistically significant difference, while 0 denotes insignificance, according to p-value of 0.05. The blank space corresponds to nan (not-a-number) values resulted as an outcome of non-variability in data in and across pair of observations.*
